# Role of Gastric Point-of-Care Ultrasound in Perioperative Management of Semaglutide

**DOI:** 10.7759/cureus.85791

**Published:** 2025-06-11

**Authors:** Mehana O Muranaka, Tony H Nguyen, Annie T Wang, Justin Cordero, Su-Jau Yang, Jasmine Felix, Jennifer L Nguyen, Antoine L Carré, Michael W Chu

**Affiliations:** 1 Anesthesiology, Kaiser Permanente School of Medicine, Pasadena, USA; 2 Anesthesiology, Southern California Permanente Medical Group, Los Angeles, USA; 3 Plastic Surgery, University of California, Riverside School of Medicine, Riverside, USA; 4 Research and Evaluation, Southern California Permanente Medical Group, Los Angeles, USA; 5 Cardiology, Southern California Permanente Medical Group, Los Angeles, USA; 6 Plastic Surgery, City of Hope, Duarte, USA; 7 Plastic Surgery, Southern California Permanente Medical Group, Los Angeles, USA

**Keywords:** clinical approaches & management, gastric point-of-care ultrasound, glp-1 receptor agonists, perioperative anesthesia, semaglutide

## Abstract

Introduction

Glucagon-like peptide-1 receptor agonists (GLP1-RAs) are used in the treatment of type 2 diabetes mellitus and obesity. A side effect of GLP1-RAs is delayed gastric emptying, which could increase the risk of pulmonary aspiration. This exploratory pilot study examines the use of ultrasound to identify high-risk patients taking GLP-1RAs before elective surgery.

Methods

A prospective study from July 2023 to February 2024 was conducted on patients who took their last weekly dose of semaglutide less than seven days before surgery. If preoperative gastric ultrasound revealed an empty stomach, surgery proceeded. Patients with residual gastric contents were rescheduled. Patient demographics, semaglutide dosage and timing, nil per os (NPO) time, and postoperative complications were reviewed. Statistical significance was set at p < 0.05.

Results

Twenty-five patients took their weekly semaglutide less than seven days before surgery. Twenty patients (80%) demonstrated empty stomachs on ultrasound and proceeded with surgery without complications. Gastric contents were found in five patients (20%), and surgery was rescheduled. Patients who took semaglutide one to three days before surgery were more likely to have residual gastric contents as compared to those who took semaglutide four to six days before surgery (p = 0.02).

Discussion

Gastric ultrasound is a useful tool that can prevent the cancellation of surgery for patients on semaglutide. Patients who took semaglutide within one to three days were more likely to have residual gastric contents compared to those who took it four to six days prior.

Conclusion

Preoperative gastric ultrasound can identify high-risk patients on semaglutide despite adequate NPO status.

## Introduction

Management of type 2 diabetes mellitus (T2DM) and weight loss has undergone a transformative shift with the introduction of glucagon-like peptide-1 receptor agonists (GLP-1RAs). Semaglutide is one of many GLP-1RA formulations that is administered subcutaneously weekly and approved by the Food and Drug Administration in 2017 for the management of T2DM and in 2021 for weight management [1]. Other more recently approved formulations include longer acting liraglutide, albiglutide, dulaglutide, tirzepatide, and shorter acting exenatide and lixisenatide [2].

The GLP-1 receptor is expressed in numerous organs such as the gastrointestinal tract, heart, liver, brain, and pancreas [3]. GLP-1 is an incretin hormone that stimulates insulin secretion after an oral glucose load. Unlike naturally occurring GLP-1, pharmacologic GLP1-RA is resistant to degradation by the dipeptidyl peptidase 4 (DPP-4) enzyme, which leads to a longer half-life [4].

Due to the various receptor sites, GLP1-RAs can improve glycemic control, reduce blood pressure, assist with weight loss without the risk of hypoglycemia, and potentially alleviate depressive symptoms in humans [5-11]. Additionally, patients on GLP1-RAs have a reduction of major adverse cardiovascular events, such as nonfatal myocardial infarction or nonfatal stroke [12-14]. In patients with heart failure with preserved ejection fraction, these patients have improved exercise function and fewer symptoms when treated with GLP1-RAs [15]. Given the efficacy and increasing accessibility of GLP1-RAs, the number of patients who are taking these medications and presenting for surgery will continue to increase.

Because GLP-1RAs have effects on gastrointestinal function by altering gastric motility, patients may have delayed gastric emptying and potential gastrointestinal disturbances [16-19]. These effects pose a unique challenge and consideration for anesthesiologists, as they can significantly impact anesthesia delivery with increased risk of aspiration of gastric contents [20-22].

In June 2023, the American Society of Anesthesiologists (ASA) released guidance on the management of perioperative patients who are currently taking this class of medications [23]. The ASA’s guidance on management of patients who are taking GLP-1RAs note that “if the patient has no GI symptoms, but the GLP-1RA medications were not held as advised, proceed with ‘full stomach’ precautions or consider evaluating gastric volume by ultrasound, if possible and if proficient with the technique” [23]. This guidance suggests that patients who did not stop the use of GLP-1RAs should be considered “full stomach,” effectively suggesting that anesthesiologists should cancel elective surgery or alternatively, do a rapid sequence induction for cases that cannot be delayed.

Following this initial guidance, in November 2023, the American Gastroenterological Association (AGA) instead advised that physicians proceed with endoscopic procedures for patients who continue GLP-1RA use if they are both asymptomatic and follow standard pre-procedure fasting protocols [24]. Similarly, the American Diabetes Association’s (ADA) recently released 2025 Standards of Care in Diabetes suggests this more personalized style of management as a mitigation strategy [25].

The ASA guidelines were updated in December of 2024 through a multisociety consensus that recommended an approach “based on shared decision-making of the patient, the prescribing care team, the proceduralist or surgeon, and the anesthesiologist” [26]. Following the ASA, AGA, and ADA statements, the consensus is to evaluate patient risk individually for GLP-1RA perioperative care management.

Gastric point-of-care ultrasound (G-POCUS) is a method used to evaluate the stomach and can therefore potentially help avoid unnecessary surgical delays. G-POCUS is a non-invasive method to assess residual gastric contents for patients prescribed GLP-1RAs [27,28]. This readily available imaging modality enables anesthesiologists to evaluate the risk of aspiration and anticipate complications specific to altered gastric physiology, thereby guiding anesthetic planning and perioperative decision-making.

This exploratory pilot study aims to explore the nuanced relationship between GLP-1RAs and perioperative management of patients receiving semaglutide within the last seven days before surgery through the use of G-POCUS. The integration of gastric ultrasound in anesthetic practice represents a promising avenue for personalized care, helping to navigate the complexities posed by GLP-1RAs and optimizing anesthetic delivery in the perioperative period. This research provides further insight into the ASA’s updated guidance for patients taking semaglutide prior to surgery. This article was previously presented as a meeting abstract at the 2024 California Society of Anesthesiologists Meeting on April 6, 2024.

## Materials and methods

A prospective study was performed after obtaining Institutional Review Board (IRB) approval from Kaiser Permanente Southern California (IRB name: Ultrasound Assessment of Gastric Contents Before Elective Surgery, approval number: 13718). Patient demographics, GLP-1RA dosage and timing, nil per os (NPO) time, type of surgery and anesthesia, gastric ultrasound images, and postoperative complications were reviewed from July 2023 to February 2024. Patients included in the study consisted of those who did not stop their last dose of GLP-1RA at least seven days before their elective surgery. GLP-1RAs included in our study consisted of the following weekly injectable medications: dulaglutide, exenatide extended release, and semaglutide. Patients who were taking daily oral and injectable GLP-1RAs, such as liraglutide and exenatide, were excluded from our study. Patients who discontinued their GLP-1RA more than a week before their scheduled surgery and patients who did not recall the timing of their last dose of GLP-1 agonist were also excluded from the study. All patients met the ASA preoperative fasting guidelines [29]. 

For all patients, gastric ultrasound was performed using the low-frequency (2-5 MHz) curved array probe (SonoSite X-Porte Ultrasound, Fujifilm Sonosite, Inc., Bothell, WA) in the parasagittal imaging plane on patients in both supine and right lateral decubitus (RLD) positions. Abdominal imaging settings were selected with an imaging depth to allow visualization of the abdominal aorta. 

**Figure 1:**
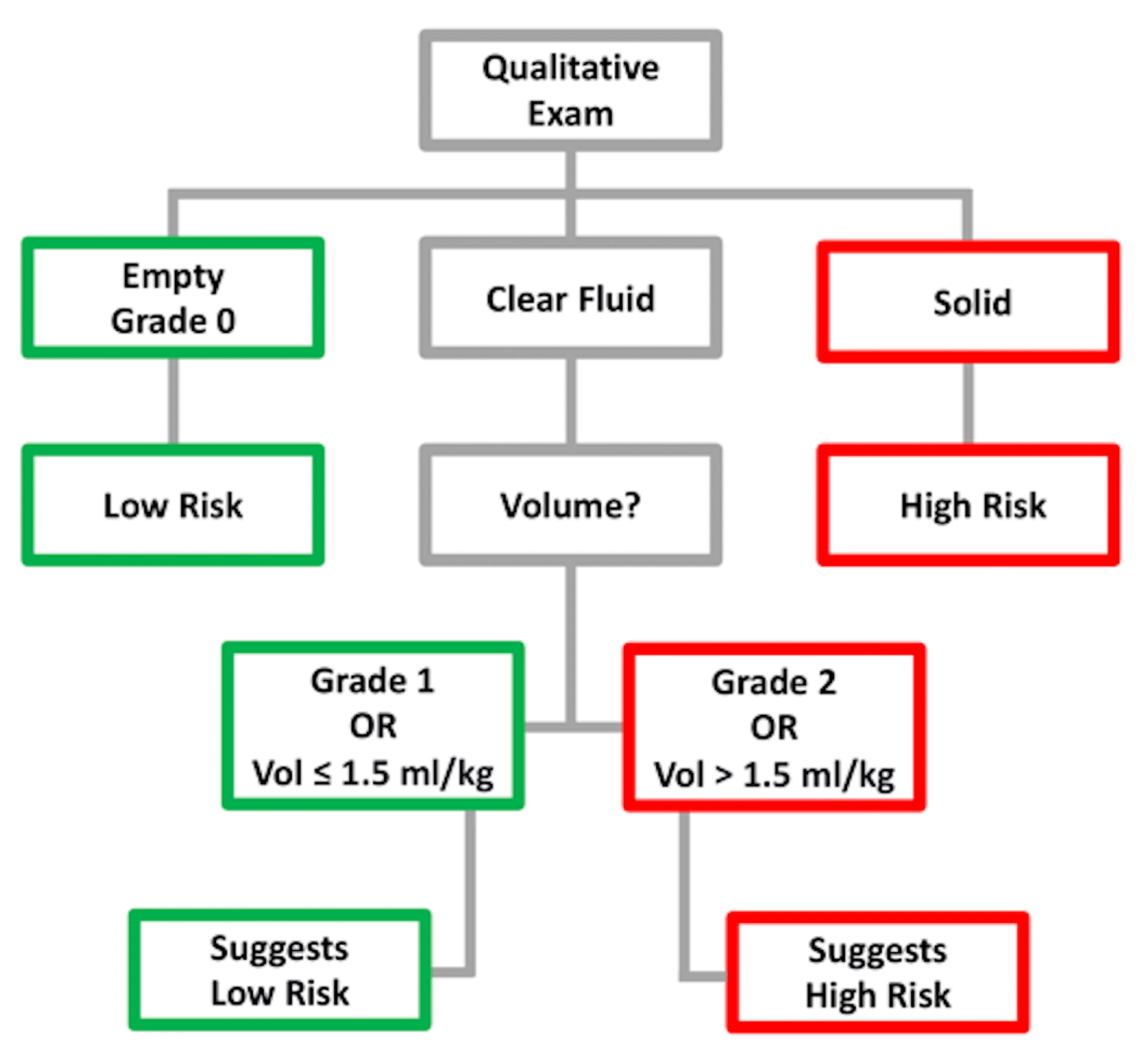
Diagnostic flowchart for interpretation and medical decision-making based on gastric point-of-care ultrasound findings Patients who had solid contents in any position and clear liquid contents with cross-sectional area (CSA) greater than 10 cm^2^ or estimated gastric volume greater than 1.5 mL/kg of the patient’s total body weight in the right lateral decubitus position were considered “full stomach.” This flow chart was adapted and used with permission from gastric ultrasound.org [32].

The ultrasound probe was placed subxiphoid and perpendicular to the patient’s abdomen, with the orientation of the probe pointing cephalad. The stomach, liver, aorta, superior mesenteric artery, and pancreas were identified by scanning the abdomen. Gastric contents and volume were assessed initially in the supine position, followed by the RLD position. Solid or thick fluid contents in any position and clear liquid contents with either cross-sectional area (CSA) >10 cm^2^ or estimated gastric volume >1.5 mL/kg of the patient’s total body weight in the RLD position were considered as “full stomach” (Figure 1) [30]. A bullseye or donut appearance of the stomach in the RLD position, clear liquid with either CSA <10 cm^2^ in the RLD position, or clear liquid with estimated gastric volume ≤1.5 mL/kg was designated as “empty stomach” [27]. Gastric ultrasound examination was performed and interpreted by qualified anesthesiologists who had prior extensive training in point-of-care ultrasound.

Statistical analysis

Statistical analysis was done using Statistical Package for the Social Sciences (IBM SPSS Statistics for Windows, IBM Corp., Version 24.0, Armonk, NY). Categorical data were analyzed using Fisher’s exact test, and numerical data were analyzed with the Wilcoxon rank sum test. Since our sample size was likely to be small, in order to compare the association between patient characteristics and contents on gastric ultrasound, we grouped the variables into two categories, instead of multiple categories for comparison (i.e., ASA 1 and 2 compared to ASA 3 and 4). A p-value of <0.05 was considered statistically significant.

## Results

Patient demographics

A total of 25 patients did not stop their GLP-1RA at least seven days before elective surgery (Table 1). The mean age of our patients was 50.8 ± 12.4 years old (range: 29-73 years old), the mean BMI was 33.8 ± 8.9 kg/m^2^ (range: 25.9-70.8 kg/m^2^), 16 of 25 patients were female (64%), and the mean ASA PS score was 2.4 (range: 1-4). Eight of 25 patients (32%) had T2DM, and one out of 25 patients (4%) had type 1 diabetes mellitus. All 25 patients (100%) took semaglutide. The average timing of the last dose of GLP-1RA was 4.4 ± 1.3 days (range: one to six days). The mean dose of semaglutide was 0.90 mg ± 0.54 mg (range: 0.25-2 mg) injected subcutaneously per week. 

**Table 1:** Patient demographics, dosage and timing of last GLP1-RA, NPO status, ultrasound findings, type of procedure, type of anesthesia, perioperative complications ASA PS: American Society of Anesthesiologists Physical Status; BMI: body mass index; CHF: congestive heart failure; CKD: chronic kidney disease; COPD: chronic obstructive pulmonary disease; DM1: type 1 diabetes mellitus; EGD: esophagogastroduodenoscopy; GERD: gastroesophageal reflux disease; GETA: general endotracheal anesthesia; GLP-1RA: glucagon-like peptide-1 receptor agonists; HLD: hyperlipidemia; HTN: hypertension; LMA: laryngeal mask airway; MAC: monitored anesthesia care; NPO: nil per os; OSA: obstructive sleep apnea; TAH-BSO: total abdominal hysterectomy and bilateral salpingo-oophorectomy; T2DM: type 2 diabetes mellitus; TIA: transient ischemic attack

Patient	Age	Gender	Past medical history	BMI (kg/m^2^)	ASA PS score	Type of GLP-1RA	Last dose of semaglutide (days prior to surgery)	Subcutaneous dose of semaglutide (mg)	NPO status per ASA guidelines	Procedure	Ultrasound findings	Type of anesthesia	Perioperative complications
1	34	M	HTN, OSA, CHF, severe morbid obesity	70.8	4	Semaglutide	1	0.5	Compliant	Urethral dilation	Full stomach	Case rescheduled	None
2	62	M	T2DM, obesity	33.8	2	Semaglutide	3	0.5	Compliant	Posterior vitrectomy	Full stomach	Case rescheduled	None
3	52	F	hypothyroid, T2DM, gastroparesis, GERD, iron deficiency anemia, thrombocytosis, HLD	27.8	2	Semaglutide	4	2	Compliant	Laparoscopic robot ventral hernia repair	Full stomach	Case rescheduled	None
4	53	F	HTN, smoker, obesity	39.1	2	Semaglutide	2	1	Compliant	Foot corn removal	Full stomach	Case rescheduled	None
5	56	F	HLD, obesity	36.1	2	Semaglutide	4	0.25	Compliant	Total knee replacement	Empty stomach	Spinal and MAC	None
6	58	M	HTN, GERD, hypothyroid, obesity	35.2	2	Semaglutide	4	0.5	Compliant	EGD and colonoscopy	Empty stomach	MAC	None
7	71	F	Asthma, COPD stage 3, HTN, T2DM, GERD, smoker	25.9	3	Semaglutide	4	1	Compliant	Perineal wide excision	Empty stomach	General anesthesia with LMA	None
8	42	F	HTN	26	2	Semaglutide	6	0.25	Compliant	Breast lump excision	Empty stomach	MAC	None
9	52	F	OSA, TIA, iron deficiency anemia, hemiplegia, obesity	39.1	3	Semaglutide	5	1	Compliant	Deep excision of soft tissue mass of hand	Empty stomach	General anesthesia with LMA	None
10	46	F	HTN, menorrhagia, osteoarthritis, obesity	36	2	Semaglutide	5	1.7	Compliant	Endometrial ablation	Empty stomach	MAC	None
11	39	F	asthma, hypothyroidism, rheumatoid arthritis, obesity	36.3	2	Semaglutide	6	1	Compliant	Laparoscopic salpingectomy	Empty stomach	GETA	None
12	49	F	HTN, hypothyroid, iron deficiency anemia, obesity	31	3	Semaglutide	4	1	Compliant	Laparoscopic robot TAH-BSO and cystoscopy	Empty stomach	GETA	None
13	36	F	asthma, HLD, endometrial cancer	29	3	Semaglutide	6	1	Compliant	Functional endoscopic sinus surgery	Empty stomach	GETA	None
14	29	M	DM1, HTN, HLD, obesity	31.9	2	Semaglutide	5	0.25	Compliant	Laparoscopic appendectomy	Empty stomach	GETA	None
15	39	F	Hidradenitis suppurativa, fibroids	28.2	2	Semaglutide	2	1	Compliant	Breast reduction	Empty stomach	GETA	None
16	73	F	HLD, asthma, urinary incontinence, obesity	33	2	Semaglutide	4	0.5	Compliant	Cystoscopy with botulinum toxin injection	Empty stomach	MAC	None
17	42	F	HLD, T2DM, GERD, obesity	32.7	2	Semaglutide	5	2	Compliant	Extracorporeal shock wave lithotripsy	Empty stomach	General anesthesia with LMA	None
18	44	F	Gilbert syndrome, T2DM, migraines	27	2	Semaglutide	6	1	Compliant	Posterior vitrectomy	Empty stomach	MAC	None
19	73	M	Spinal stenosis, OSA, interstitial lung disease, GERD, Raynaud's syndrome, prediabetes	26.4	2	Semaglutide	5	2	Compliant	Carpal tunnel release	Full stomach	Case rescheduled	None
20	41	F	Carpal tunnel syndrome, renal calculus, homozygous methylenetetrahydrofolate reductase mutation	28.5	2	Semaglutide	5	0.5	Compliant	Ureteroscopy with laser lithotripsy	Empty stomach	General anesthesia with LMA	None
21	62	M	T2DM, sepsis, gangrene of foot, acute kidney injury, obesity	34.4	3	Semaglutide	4	0.5	Compliant	First ray amputation	Empty stomach	MAC	None
22	50	M	HLD, irritable bowel syndrome, obesity	36.7	2	Semaglutide	6	1	Compliant	Knee arthroscopy with meniscal repair	Empty stomach	GETA	None
23	45	F	Asthma, HTN, HLD, migraine, OSA, angioedema	30.2	3	Semaglutide	5	0.5	Compliant	TAH-BSO	Empty stomach	GETA	None
24	70	M	HTN, HLD, T2DM, Jehovah’s witness, prostate cancer, CKD stage 4, hyperparathyroidism, CHF	29.7	3	Semaglutide	5	1	Compliant	Laparoscopic peritoneal dialysis catheter insertion	Empty stomach	GETA	None
25	53	M	HTN, HLD, T2DM, cluster headaches, OSA	40.8	3	Semaglutide	5	0.5	Compliant	Middle phalanx osteotomy and pinning	Empty stomach	MAC with supraclavicular nerve block	None

Ultrasound findings

**Figure 2:**
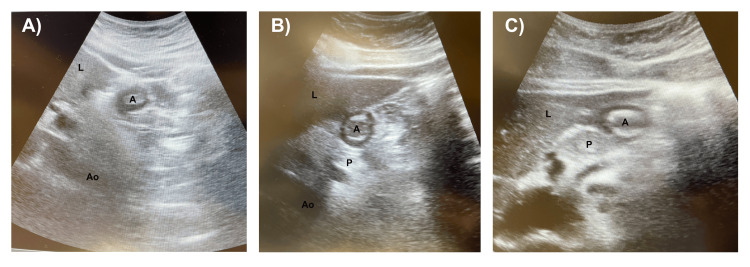
Gastric point-of-care ultrasound demonstrating the empty stomach of three different patients (A, B, and C) The images of these patients were obtained in the right lateral decubitus position to increase the sensitivity of the exam. Pertinent structures were identified, such as the liver (L), gastric antrum (A), aorta (Ao), and pancreas (P). An empty stomach has a “bulls-eye” appearance, indicative of a small or collapsed antrum with a distinct circumferential stomach wall, as seen in A, B, and C.

**Figure 3:**
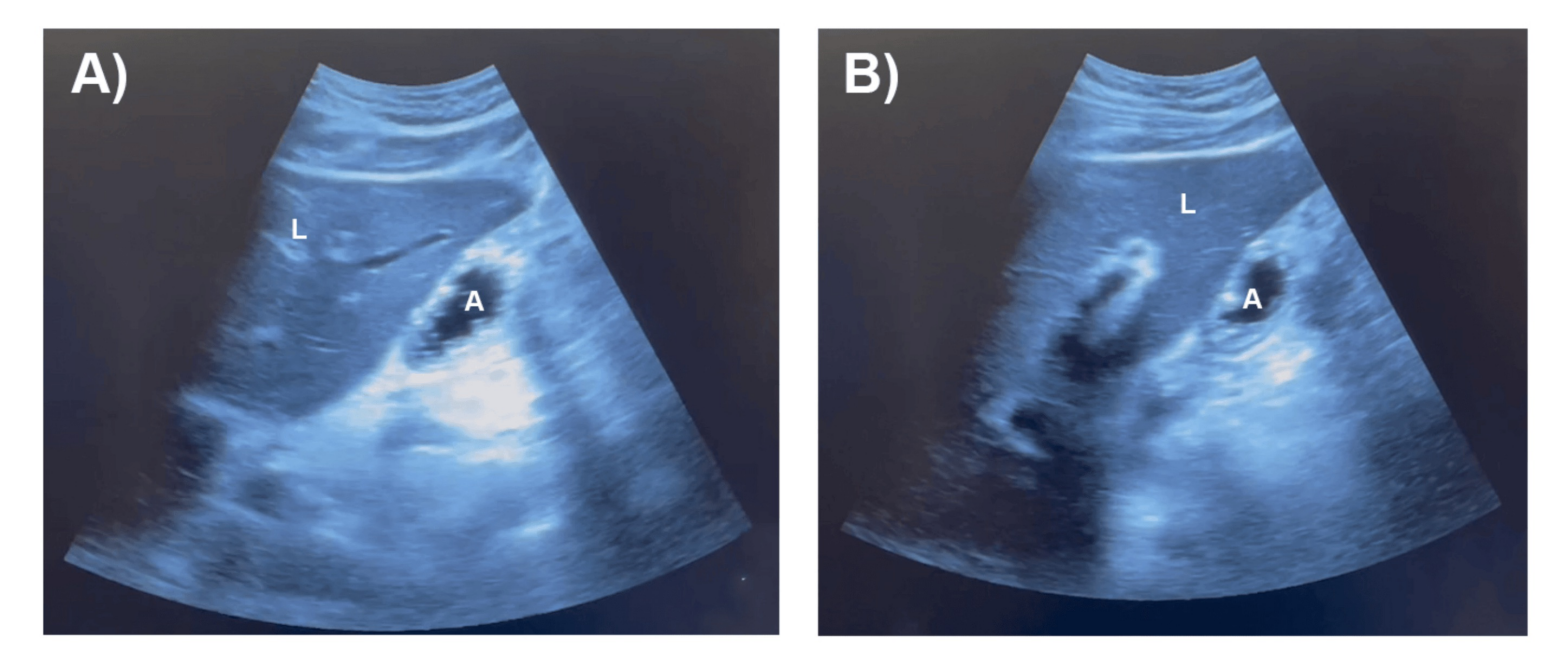
Gastric point-of-care ultrasound demonstrating clear liquids in the antrum in the right lateral decubitus position Pertinent structures were identified, such as the liver (L) and gastric antrum (A). Clear liquids appear homogenous and hypoechoic in the antrum (A, B). After identification of clear liquids, the cross-sectional area is measured to aid in the calculation of the estimated gastric volume. Both images (A and B) were taken from the same patient.

**Figure 4:**
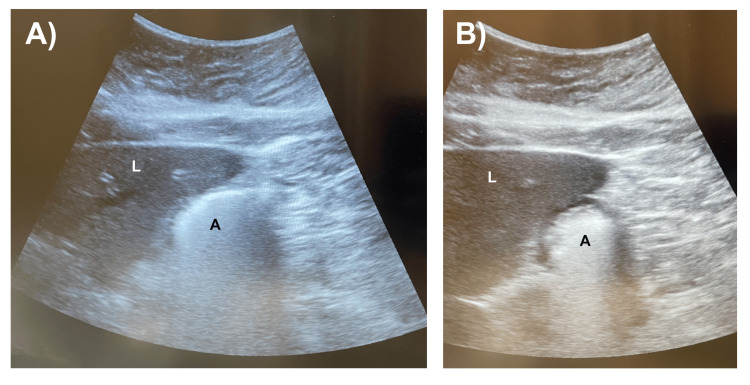
Gastric point-of-care ultrasound demonstrating solid gastric contents in the antrum in the right lateral decubitus position (A) and supine position (B) of the same patient Pertinent structures were identified, such as the liver (L) and gastric antrum (A). Solid gastric contents appear heterogeneous and hyperechoic (bright) in the antrum. Structures distal to the solid contents (A, B) may not be visible due to poor penetration of the ultrasound waves, also known as “shadow artifact.”

All 25 patients (100%) adhered to the ASA NPO guidelines. Twenty of 25 (80%) patients had an “empty stomach” on ultrasound imaging (Figures 2A-2C), and all of these patients proceeded with surgery the same day without complications. Nineteen of the 20 patients (95%) with empty stomach had grade 0 antrum with the “bullseye” appearance in both the supine and RLD positions. One of the 20 patients (5%) with an empty stomach had clear liquids in the stomach on ultrasound imaging, and the estimated gastric volume was calculated by measuring the CSA (7.08 cm^2^) and using the following equation: 27 + (14.6* CSA) - (1.28 * age) (Figures 3A-3B). The estimated gastric volume was 66 mL, which was less than 1.5 mL/kg of the patient’s total body weight. Therefore, this patient was considered to have an empty stomach. Five of the 25 (20%) patients had a “full stomach” on ultrasound (Figures 4A-4B), and surgery was rescheduled. Three of the 20 patients (15%) with an “empty stomach” on ultrasound that proceeded with surgery were first-start cases, and all three first-start surgeries (100%) started on time without any delay from performing the gastric point-of-care ultrasound. 

Statistics

Patients who took their GLP-1RA one to three days before their elective surgery were significantly more likely to have a full stomach on gastric ultrasound as compared to patients who took their GLP-1RA four to six days before surgery (p = 0.02) (Table 2). Three of the four patients (75%) who took their GLP-1RA one to three days before surgery were found to have a full stomach on ultrasound. Two of the 21 (10%) patients who took their GLP-1RA four to six days before surgery were found to have a full stomach on ultrasound (Table 2). Additionally, patients who took their last dose of GLP-1RA four to six days before surgery were 28 times more likely to have an empty stomach as compared to patients who took their last dose of GLP-1RA one to three days before surgery (OR: 28.5, 95% CI: 1.93-420.41, p = 0.01) (Table 3). There were no significant differences for those who took a smaller dose of GLP-1RA (0 to 0.5 mg) as compared to those who took a higher dose of GLP-1RA (>0.5 mg). There were no other statistically significant associations between patient demographics (age, gender, diabetes, BMI, and ASA status) and the presence of a full or empty stomach (Table 2). Since our sample size (n = 25) was too small to conduct multivariate logistic regression, our data only present the results from univariate logistic regression.

**Table 2:** Patient characteristics of those with full stomach versus empty stomach on ultrasound For continuous variables (i.e., mean age), the data have been represented as mean ± standard deviation. For categorical variables (i.e., sex, presence of diabetes mellitus, etc.), the data have been represented as N (%). P < 0.05 is considered statistically significant. ASA: American Society of Anesthesiologists, BMI: body mass index, T1DM: type 1 diabetes mellitus, T2DM: type 2 diabetes mellitus

Characteristic	Empty stomach on ultrasound (n = 20)	Full stomach on ultrasound (n = 5)	p-value
Mean age	49.9 ± 12.1	54.8 ± 14.4	0.43
Sex			0.31
Female	14 (88%)	2 (12%)	
Male	6 (67%)	3 (33%)	
Diabetes			1.00
T1DM and T2DM	7 (78%)	2 (22%)	
No diabetes	13 (81%)	3 (19%)	
Last dose of semaglutide (days prior to surgery)			0.02
1-3 days	1 (25%)	3 (75%)	
4-6 days	19 (90%)	2 (10%)	
Dose of semaglutide (mg)			1.00
>0.5 mg	11 (79%)	3 (21%)	
0-0.5 mg	9 (82%)	2 (18%)	
BMI (kg/m^2^)			1.00
≤30	7 (78%)	2 (22%)	
>30	13 (81%)	3 (19%)	
ASA score			0.62
1 and 2	12 (75%)	4 (25%)	
3 and 4	8 (89%)	1 (11%)	

**Table 3:** Odds ratio of risk of empty stomach as compared to full stomach The data are represented as odds ratios (ORs) with the corresponding 95% confidence intervals. P-value < 0.05 is considered statistically significant. ASA: American Society of Anesthesiologists; BMI: body mass index; CI: confidence interval; OR: odds ratio; Ref: reference; T1DM: type 1 diabetes mellitus; T2DM: type 2 diabetes mellitus

	Unadjusted			
	OR	95% CI		p-value
		Lower	Upper	
Age, per one year increase	0.97	0.89	1.05	0.42
Sex				
Male	Ref			
Female	3.50	0.46	26.62	0.23
Diabetes				
No DM	Ref			
T1DM and T2DM	0.81	0.11	6.04	0.84
Last dose of semaglutide (days prior to surgery)				
1-3 days	Ref			
4-6 days	28.49	1.93	420.41	0.01
Dose of semaglutide (mg)				
0-0.5 mg	Ref			
>0.5 mg	1.23	0.17	9.02	0.84
BMI (kg/m^2^)				
≤30	Ref			
>30	1.24	0.17	9.25	0.84
ASA score				
1 and 2	Ref			
3 and 4	2.67	0.25	28.44	0.42

## Discussion

The use and popularity of GLP-1RA continue to grow due to its impact on weight loss, glycemic control, and reduction in cardiovascular events. Despite its benefits, the side effects of GLP1-RA use can impact perioperative management. There are risks for patients who stop their GLP-1RA use prior to surgery, as well as if they continue. For patients who discontinue use, there is evidence of associated risks such as reduced glycemic control and related adverse surgical outcomes [31]. Inevitably, there will be more patients who will be taking semaglutide within seven days prior to their surgery. Given the potential for significant morbidity and a 57% mortality rate directly related to pulmonary aspiration if it occurs [32], anesthesiologists will need to find ways to mitigate risk while minimizing delays in surgical care. G-POCUS is a safe, non-invasive imaging modality that can define the presence of an “empty” or “full” stomach [27].

Previous research has found that the use of GLP1-RAs increased the risk of residual gastric content [17,18] and specifically associated semaglutide with “full” stomach even after appropriate overnight fasting [33]. 

In addition to prior studies, our exploratory pilot examined the utility of ultrasound imaging in the management of patients undergoing elective surgery who did not discontinue their weekly GLP-1RAs within seven days, as recommended by the 2023 ASA perioperative guidance on GLP-1RA use [23]. In 2023, without a gastric ultrasound, the ASA guidance suggested that these patients should be canceled under the assumption that they may have “full” stomachs. Our primary finding was that by performing a gastric ultrasound, 80% (20 out of 25) of our patients were able to safely proceed with elective surgery without postoperative complications. Not only can gastric ultrasound improve patient care by potentially minimizing the risk of aspiration, but it can also minimize surgery cancellations, which can improve patient satisfaction [34,35].

In addition, our research adds to the growing literature delineating the risks of GLP-1RA use perioperatively [19-22]. Despite following the NPO guidelines for preoperative fasting [29], five out of 25 of our patients (20%) who continued their GLP1-RA within the last seven days had solid gastric contents, resulting in their elective surgeries being rescheduled. These data lend additional support for the ASA’s recommendation of G-POCUS for evaluation of patient risk.

Our study also found that patients who had taken their last dose of semaglutide one to three days before surgery were more likely to have a “full stomach” compared to those who took their last dose of semaglutide four to six days prior to surgery (p = 0.02). This suggests that semaglutide use within three days of surgery increases the risk of a “full stomach.” Having this information may help anesthesiologists better risk-stratify their patients and standardize care. We did not find statistical significance with patient characteristics, such as age, gender, diagnosis of type 1 or 2 diabetes mellitus, BMI, and ASA status, when evaluating the presence of a “full stomach.” 

Although this study was designed to include all GLP-1RAs, all of our patients who met the inclusion criteria took semaglutide. A potential explanation could be that the majority of patients at this hospital were overwhelmingly prescribed semaglutide compared to other GLP-1RAs. Out of the 51,864 patients at our hospital who were prescribed GLP-1RAs, 50,621 patients (97.6%) were prescribed semaglutide. The remainder of patients on GLP-1RAs were prescribed dulaglutide (1,241 out of 51,864 patients, or 2.4%) and exenatide (two out of 51,864 patients, or 0.004%). This uneven distribution may account for the exclusive use of semaglutide within our cohort. 

We acknowledge that the effectiveness of G-POCUS for perioperative management of semaglutide is contingent on physician technique and ultrasound interpretation. Therefore, our findings suggest that recent graduates of anesthesiology residency programs and practicing anesthesiologists should learn the skill of using ultrasound to precisely look for gastric contents. This goal aligns with the recommendations by the Accreditation Council for Graduate Medical Education (ACGME) and the American Board of Anesthesiologists (ABA) [36,37]. To further the goal of mastering G-POCUS, the APPLIED Exam Objective Structured Clinical Examination (OSCE) requires that anesthesiologists will need to demonstrate the use of ultrasound to assess gastric content and volume starting in 2024 [37].

However, it is important to note that clinical practice often lags behind practice guidelines due to numerous barriers. Similar to the introduction of ultrasound for the placement of central venous catheters, related adoption barriers are to be expected. For instance, in surveys that evaluated the attitudes and beliefs of anesthesiologists on the use of ultrasound for central venous catheter placement, there was a significant gap between clinical practice and the recommended practice guidelines [38]. Nonetheless, with further research and education, practice guidelines eventually became best practice as more providers obtain this skill set. Additionally, due to the sharp increase in GLP-1RA use for T2DM and obesity, there is a tangible incentive for standardized management of semaglutide perioperative care with the implementation of G-POCUS. 

As previously mentioned, competency in performing and interpreting gastric ultrasound is operator-dependent. To minimize variations in technique and interpretation, the physicians who performed gastric ultrasound worked in pairs to ensure consistency. If another physician was not available, images were reviewed to ensure interprovider reliability. 

The physicians who performed gastric ultrasound used both quantitative and qualitative measurements. Qualitative measurements were used for solid gastric contents. Prior research that evaluated anesthesiologist G-POCUS learning curves was able to estimate an average number of 33 cases required to achieve a 95% success rate for qualitative measurements [39]. Sufficient exposure could therefore be a method to ensure consistent interprovider qualitative accuracy in future practice.

Quantitative measurements were used for liquid contents by calculating the CSA to determine if the estimated gastric volume was ≥1.5 mL/kg in the RLD position, which would be considered a full stomach. Quantitative measurements could potentially introduce interprovider variability depending on skill level and technique. 

The goal of this exploratory study was to analyze the utility of gastric ultrasound in patients who did not discontinue their weekly GLP-1RAs and the rate of surgical cancellation. Although this primary goal was achieved, secondary goals could not be evaluated due to the small number of patients and their effect on statistical significance. Future studies should be conducted with larger sample sizes to further elucidate associated time intervals that lead to a higher risk of “full stomach.” Additionally, factors that could affect the likelihood of a full stomach on a gastric ultrasound could be investigated.

Possible factors to be evaluated in future studies include the timing between the last meal and the gastric ultrasound examination, the timing between the last clear liquid and the gastric ultrasound examination, the type of food last consumed, and acute versus chronic use of GLP1-RAs.

Gastric ultrasound findings can be influenced by a variety of patient factors, such as diabetes mellitus, body mass index, and gastric anatomy. Understanding these factors is crucial for the correct interpretation of ultrasound images and, therefore, could affect the accuracy of G-POCUS interpretations in this study.

Patients who met the criteria to be included in this study took a formulation of semaglutide. Given that each type of GLP-1RA has unique pharmacokinetic/pharmacodynamic profiles [40], the results may differ depending on the formulation. Therefore, additional studies should be conducted to determine whether other GLP-1RAs yield differing results.

Future challenges

Unfortunately, as more providers become aware of the 2023 and recently updated 2024 ASA guidance on preoperative management of patients on GLP-1RAs, it will become increasingly difficult to find patients who did not discontinue their weekly GLP-1RA. This may be a result of provider motivation to inform their patients of potential surgical cancellation with continued GLP-1RA use and changing hospital policies. Future studies at institutions that have not adopted a global policy on GLP-1RA perioperative use have a narrow window of opportunity to evaluate additional factors listed and further delineate risk factors for patients who continue to use GLP-1RAs preoperatively.

## Conclusions

Gastric ultrasound is a useful preoperative tool that can prevent cancellation of elective surgery for those who have taken semaglutide within the last seven days. Patients who took semaglutide within one to three days were more likely to have residual gastric contents compared to those who took it four to six days prior. Preoperative gastric ultrasound can identify high-risk patients on semaglutide despite adequate NPO status, which lends further research to support the ASA’s guidance for patients on GLP-1RAs perioperatively. Further research is needed to understand the risks for patients taking semaglutide prior to surgery.
